# Wide field imaging energy dispersive X-ray absorption spectroscopy

**DOI:** 10.1038/s41598-019-54287-8

**Published:** 2019-11-27

**Authors:** Peng Qi, Nazanin Samadi, Mercedes Martinson, Olena Ponomarenko, Bassey Bassey, Ariel Gomez, Graham N. George, Ingrid J. Pickering, L. Dean Chapman

**Affiliations:** 1 Division of Biomedical Engineering, University of Saskatchewan, Saskatoon, Canada; 2 Department of Physics and Engineering Physics, University of Saskatchewan, Saskatoon, Canada; 3 Canadian Light Source, Saskatoon, Canada; 4 Molecular and Environmental Science Group, Department of Geological Sciences, University of Saskatchewan, Saskatoon, Canada; 5 Department of Chemistry, University of Saskatchewan, Saskatoon, Canada; 6 Anatomy, Physiology and Pharmacology, University of Saskatchewan, Saskatoon, Canada

**Keywords:** X-ray tomography, Silicon photonics, Imaging and sensing

## Abstract

A new energy dispersive X-ray absorption spectroscopy (EDXAS) method is presented for simultaneous wide-field imaging and transmission X-ray absorption spectroscopy (XAS) to enable rapid imaging and speciation of elements. Based on spectral K-Edge Subtraction imaging (sKES), a bent Laue imaging system diffracting in the vertical plane was developed on a bend magnet beamline for selenium speciation. The high flux and small vertical focus, forming a wide horizontal line beam for projection imaging and computed tomography applications, is achieved by precise matching of lattice plane orientation and crystal surface (asymmetry angle). The condition generating a small vertical focus for imaging also provides good energy dispersion. Details for achieving sufficient energy and spatial resolution are demonstrated for both full field imaging and computed tomography in quantifying selenium chemical species. While this system has lower sensitivity as it uses transmission and may lack the flux and spatial resolution of a dedicated focused beamline system, it has significant potential in rapid screening of heterogeneous biomedical or environmental systems to correlate metal speciation with function.

## Introduction

X-ray Absorption Spectroscopy (XAS) is one of the methods used for imaging and speciation of heavier elements in biological samples^1^. It involves measuring absorption as a function of energy near and above the absorption edge (e.g. K-edge) of an element^2^. XAS is often divided into two distinct regions: X-ray Absorption Near Edge Structure (XANES)-region from 0 up to about 50 eV above the absorption edge and Extended X-ray Absorption Fine Structure (EXAFS)-region from 50 to 1000 eV above the absorption edge^1,2^. Conventional x-ray and synchrotron radiation sources have been used for XAS. The advantages of using synchrotron sources over conventional x-ray sources are the continuous spectrum, high flux and brightness, and small source size and beam divergence^3^.

The classical method of performing XAS, which is mostly used, is to scan mechanically through the required energy range using flat double-crystal monochromators. Most XAS measurements of dilute systems are achieved by detecting fluorescence from the element of interest. The prevalence of using fluorescence is because of the high sensitivity which requires an energy scanning method so that the excitation energy and fluorescence can be correlated^4^.

In Energy Dispersive XAS (EDXAS), a bent crystal monochromator is typically used to provide a focused x-ray beam that contains all the energies required to make an XAS measurement^4^. Bent crystal monochromators in either the Bragg (reflection) or Laue (transmission) geometry have been developed for EDXAS with the former being the first type to be developed, and the more frequently used^5–7^. The Laue geometry may require a thin crystal to avoid significant photon loss through the crystal depending on the x-ray energy chosen. High stability during measurements, acquisition of the spectral data in a very short time (few microseconds), and the simultaneous collection of the whole x-ray absorption spectrum are the reported advantages of EDXAS compared to the classical method^8^.

There are few instances of the Laue geometry being used for dispersive XAS systems^9–12^. One of the impediments of using this geometry is the energy blurring that occurs due to “diffraction” in depth through the crystal thickness because of the bending of the crystal, which results in the Borrmann fan^13^ on the exit surface of the crystal. The energy dispersive properties essential to the success of XAS are degraded by this energy blurring.

All dedicated EDXAS beamlines use the horizontal fan of radiation to provide an energy range for XANES and EXAFS and focus the energy dispersed beam onto the sample. These systems provide high intensity, small focus size and stability for interrogating systems rapidly or in complex environments. However, since the focus sizes on both the vertical and horizontal dimension are small, speciation imaging for larger samples requires mechanically scanning the sample in both dimensions, thus the imaging efficiency is largely limited.

There is a need for EDXAS systems that are more amenable to full field imaging applications of specimens such as plants and animals. Based on previous experience with spectral K-Edge Subtraction imaging (sKES)^14–16^, a bent Laue imaging system was developed on a bend magnet beamline, which diffracts in the vertical plane and preserves the horizontal dimension of the beam. This system forms a wide horizontal line beam with high flux and A small vertical focal size at the sample location, which sets the spatial resolution perpendicular to the line beam. In addition, the system has sufficient energy dispersion and resolution for selenium (Se) speciation and is completely compatible with full field imaging or computed tomography. This is achieved when a proper matching condition involving the relation between the orientation of the lattice planes and the crystal surface is met (asymmetry angle). Remarkably, the condition that provides very small focal conditions for imaging also provides very good energy dispersion^14^.

Though the sensitivity is limited due to the use of transmission XAS, a potential application is for rapid screening of biological/biomedical systems for the role of speciation in function. For the initial application of this approach to speciation imaging, selenium was chosen because of its biological importance and the variety of the oxidation states it presents.

Selenium is essential at trace levels in the diet for human and animal health^1,17,18^. Though toxic at high concentrations^19^, it has been reported that up to 1 billion people worldwide are selenium deficient^20^ and several studies suggest Se supplementation can reduce the risk of some cancers^21–25^. Some common species of selenium that demonstrate its different oxidation states are selenate (SeO_4_^2−^), selenite (SeO_3_^2−^) and selenide (Se^2−^). As a means of adaptation for survival in Se-rich soil, some plants, referred to as Se hyperaccumulators (e.g., *Astragalus bisulcatus* and *Stanleya pinnata*), are known to accumulate Se in excess of 1% plant dry weight^26,27^. Due to the importance of Se, several studies using spatial imaging, speciation, quantification and distribution of Se in plants and animals have been undertaken^17–19,28–32^.

**Figure 1:**
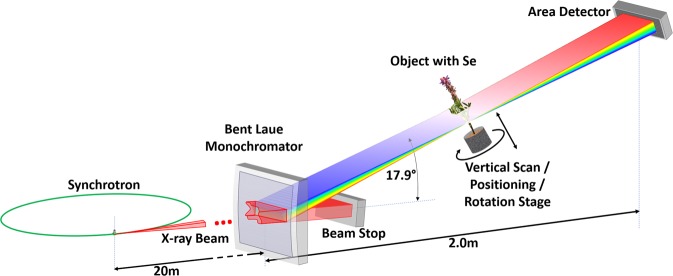
Schematic (**a**) of the bent Laue energy dispersive system for speciation imaging application. On the top left (**b**) shows the crystal orientation before being bent. There is a 5-degree angle between the normal of the crystal surface and the diffraction planes.

For these reasons, a Se specific speciation imaging system based on a bent Laue monochromator was developed. By proper choice of crystal diffraction planes and asymmetry angle of those planes in relation to the crystal surface, an energy dispersive wide field imaging system was designed and shown schematically in Fig. 1. The key to achieve good spatial and energy resolution is the relationship between the energy or Bragg angle for the lattice planes, and the relative angle between the lattice planes and the crystal surface (the asymmetry angle). Details are contained in the Method section.

The feasibility and performance of sKES for speciation imaging of selenium was demonstrated and compared with standard XAS in terms of acquisition time and energy resolution.

## Results

### Feasibility test

**Figure 2:**
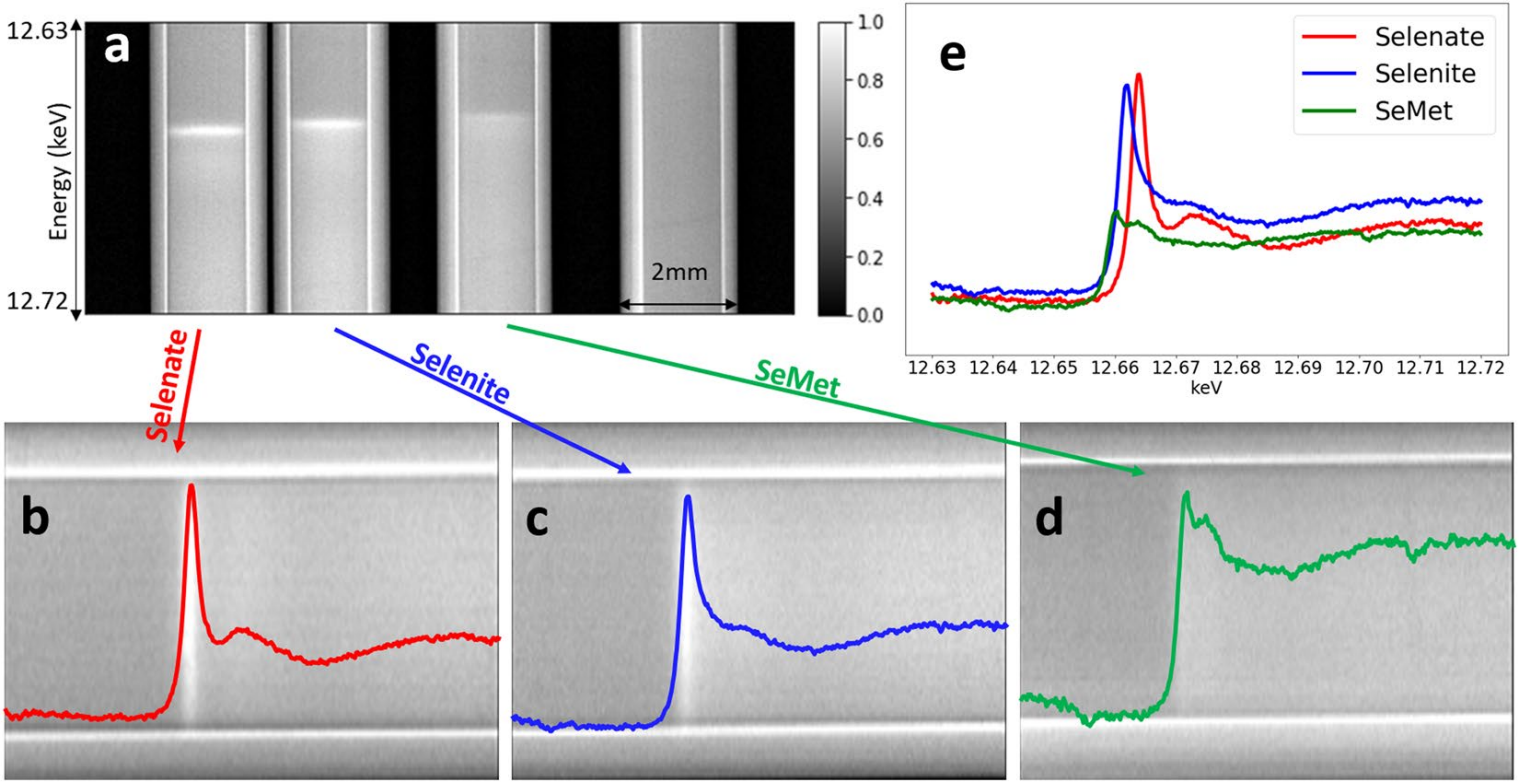
(**a**) A projection image of Na_2_SeO_4_ (Selenate), Na_2_SeO_3_ (Selenite), selenomethionine (SeMet) solutions and water in separate tubes (from left to right) with 100 ms exposure time. Figure (**b–d**) show the three tubes with Se compounds rotated so the horizontal axis is energy with superimposed (80 horizontally adjacent spectra) plots of the absorption spectra; each plot is color coded specifically to a compound. The absorption edge jump can be clearly observed in each tube and plot. (**e**) The absorption profiles from (**b–d**) superimposed for comparison.

An example of a line projection image of a series of tubes of Se compounds or water at the system focus is shown in Fig. 2a. The vertical direction in the image is photon energy increasing from top to bottom covering an energy range of approximately 90 eV. The sample included 100 mM solutions of Na_2_SeO_4_ (Selenate), Na_2_SeO_3_ (Selenite) and selenomethionine (SeMet) prepared with bicine buffer and placed in 2 mm internal diameter nylon tubes. As noted in the caption, the absorption edge is clearly seen, and plots of the measured absorption are shown over the tubes (Fig. 2b–d). The plots when compared show the variation in edge location and structure due to speciation (Fig. 2e). The acquisition time of the projection image with the Hamamatsu AA60 + ORCA Flash 4.0 detector is 100 ${\rm{ms}}$.

**Figure 3:**
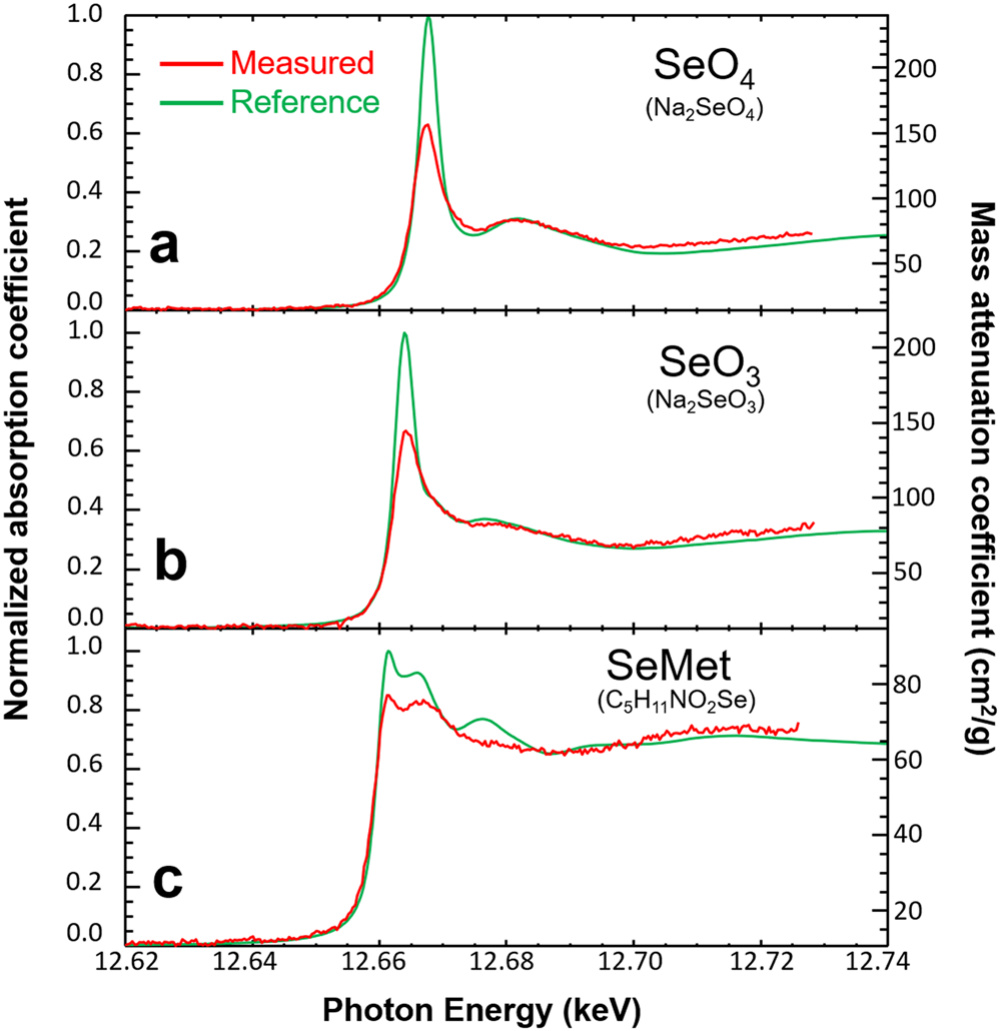
Spectrum comparison for experiment data (Measured) and traditional XAS measurement (Reference) for (**a**) Na_2_SeO_4_, (**b**) Na_2_SeO_3_ and (**c**) Selenomethionine.

The measured absorption profiles (Measured) were compared to high energy resolution measurements (Reference) using a dedicated XAS beamline with a double crystal monochromator^33^ and are shown in Fig. 3. The curves in the plot demonstrate the relation between mass absorption coefficient (cm^2^/g) and photon energy (keV) for the Measured and Reference profiles. A 1.5 eV convolution is required to obtain a reasonable match between the Measured and Reference data, indicating the reduced energy resolution of the bent Laue system. This is expected because of the inherently poorer energy resolution of the bent Laue system. The signal to noise ratio in the measured data can be improved by increased acquisition time and signal averaging. In addition, the detector used in these experiments is an imaging detector and is not particularly well suited for spectroscopy measurements. A pixel array detector with the ability to photon count above a threshold would improve significantly the signal-to-noise.

A sample was prepared for Computed Tomography (CT) imaging that include the 100 mM solutions of the Se compounds as well as a natural *Astragalus bisulcatus* seedpod which was collected on the Saskatchewan River bank in Saskatoon, SK, Canada. CT images were acquired with the solution tubes placed in a circle with the seedpod placed in the center. Each CT slice contained 900 projections (180 degrees in 0.2-degree steps). The average acquisition time per slice was 12 minutes which is significantly less compared to a typical XAS scan reported to be hours for one slice depending on the size of the desired area for analysis^34^. While the total energy range of the beam at the focus is about 500 eV, the energy range was cropped during data collection to be about 180 eV centered at 12.658 keV (Se K-edge) for analysis. The cropping of the energy range can either be done physically with apertures in the beamline or post data collection by limiting the energy range during the data analysis. Both approaches have advantages with the use of apertures allowing reduction of dose and the use of software cropping allowing the most flexibility for data analysis. In practice, both approaches are used in the data presented.

**Figure 4:**
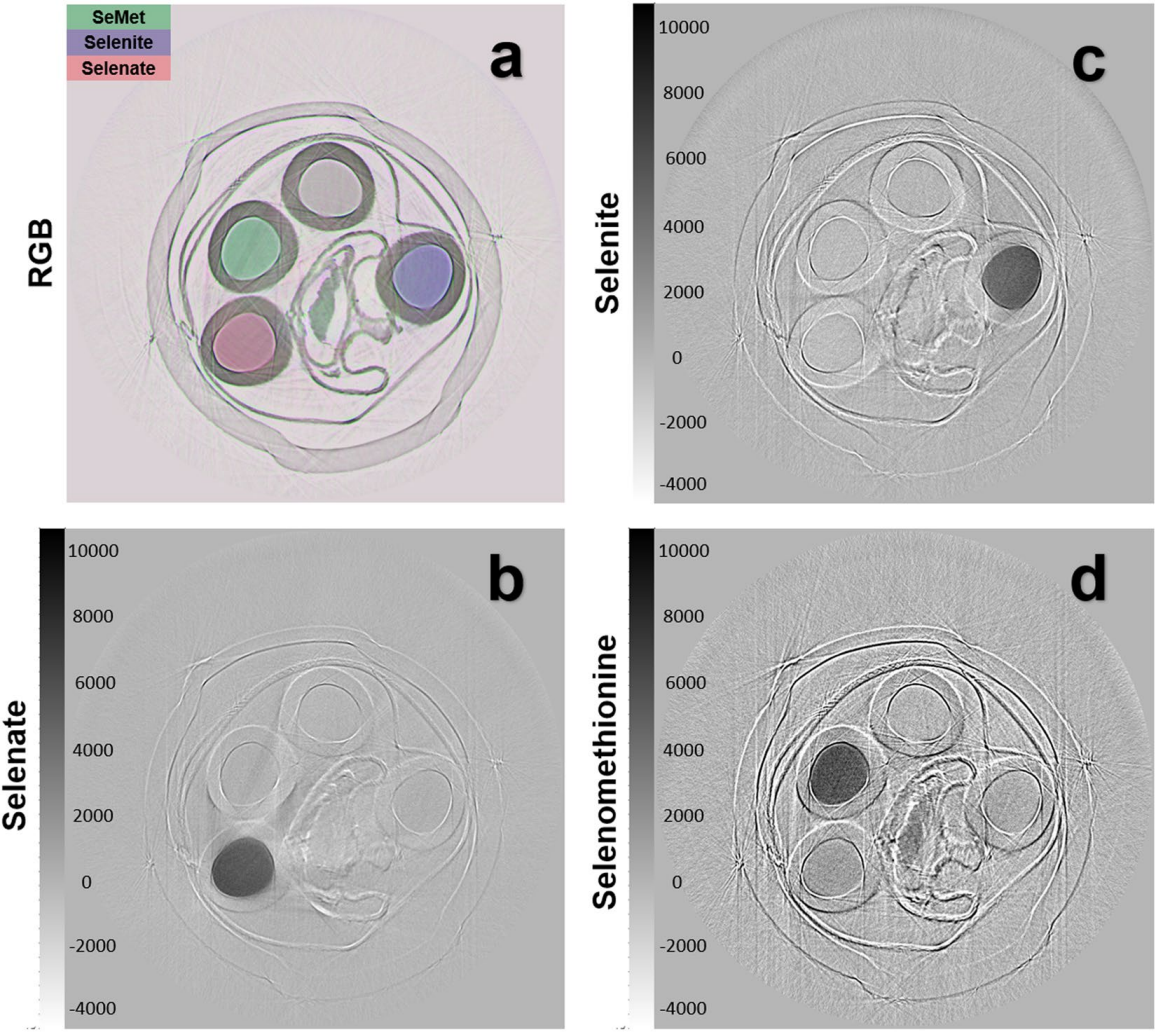
CT reconstruction of Se compound solutions. (**a**) A composite image of the reconstructed data with the water equivalent concentration shown as gray, SeMet concentration as green, selenite as blue and selenate as red. The individual reconstructed selenium compound concentrations are shown in (**b**) selenate, (**c**) selenite and (**d**) selenomethionine.

The spectral transmission data acquired with the focused beam through the object is converted into projected concentration values using a least squares algorithm fit to the Reference data. Again, a 1.5 eV convolution to the Reference data is required before the fitting. The projected concentration line images at each angular setting then form sinograms that are reconstructed into section images using a parallel beam ramp-filter back-projection algorithm. The section images are shown in Fig. 4.

**Figure 5:**
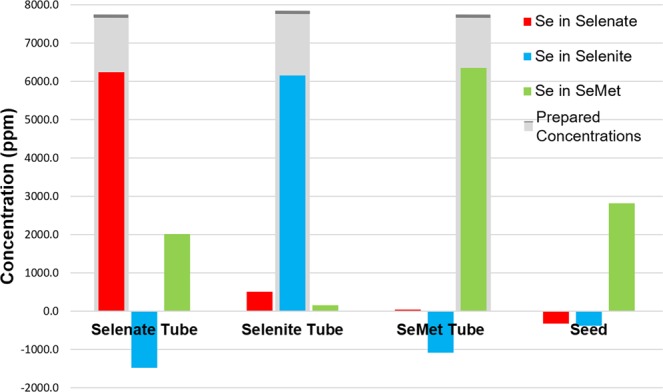
Measured (colored bars) and prepared (gray bars) concentration of compounds from reconstructions in Fig. 4 for selenate (Na_2_SeO_4_), selenite (Na_2_SeO_3_), SeMet solutions in separate tubes, and the tube with the *Astragalus bisulcatus* seedpod.

As can be seen from the CT reconstruction, all of the tubes with specific selenium compounds are distinguished from each other. Figure 5 shows the measured concentration of every compound in the sample solutions, with a comparison to the actual concentration prepared. For example, in the Selenate tube, the solution contains 100 mM Na_2_SeO_4_, and no Na_2_SeO_3_ or SeMet. The measured concentration is comparable to the prepared concentration. Measurements of Selenite and SeMet solutions show better correspondence to the prepared concentration. Note the presence of negative concentration values in the figure for the selenite compound and the presence of false positive values for the selenate solution sample. The negative values are unphysical. A non-negative least squares algorithm can be used to prevent these values, but their presence indicates the lower detectability level for the system and data acquired. The presence of false values could be due to improper normalization, improper matching of the Reference standards resolution compared with that of the Laue configuration, or noise in the imaging system. Better thermal cooling and crystal polishing in future experiments is expected to improve the signal to noise level. Furthermore, measuring standard materials with the Laue system for use in the data analysis can avoid matching resolutions between systems.

Remarkably, significant amount of SeMet (7 mg/cm^3^) is detected in the seedpod reconstruction images. The dominant form of selenium compounds in the seed of *Astragalus bisulcatus* was reported to be Se-methylselenocysteine^19^. Because the local structural environments of Se-methylselenocysteine and SeMet are similar, each bound to one methyl and one other aliphatic carbon, their XAS are almost the same in terms of edge energy and near edge structure^19^; the measured concentration of SeMet thus is expected to represent the Se-methylselenocysteine contained in the seed and is evidence for the application of the method for speciation imaging in plant samples.

### Concentration sensitivity test

**Figure 6:**
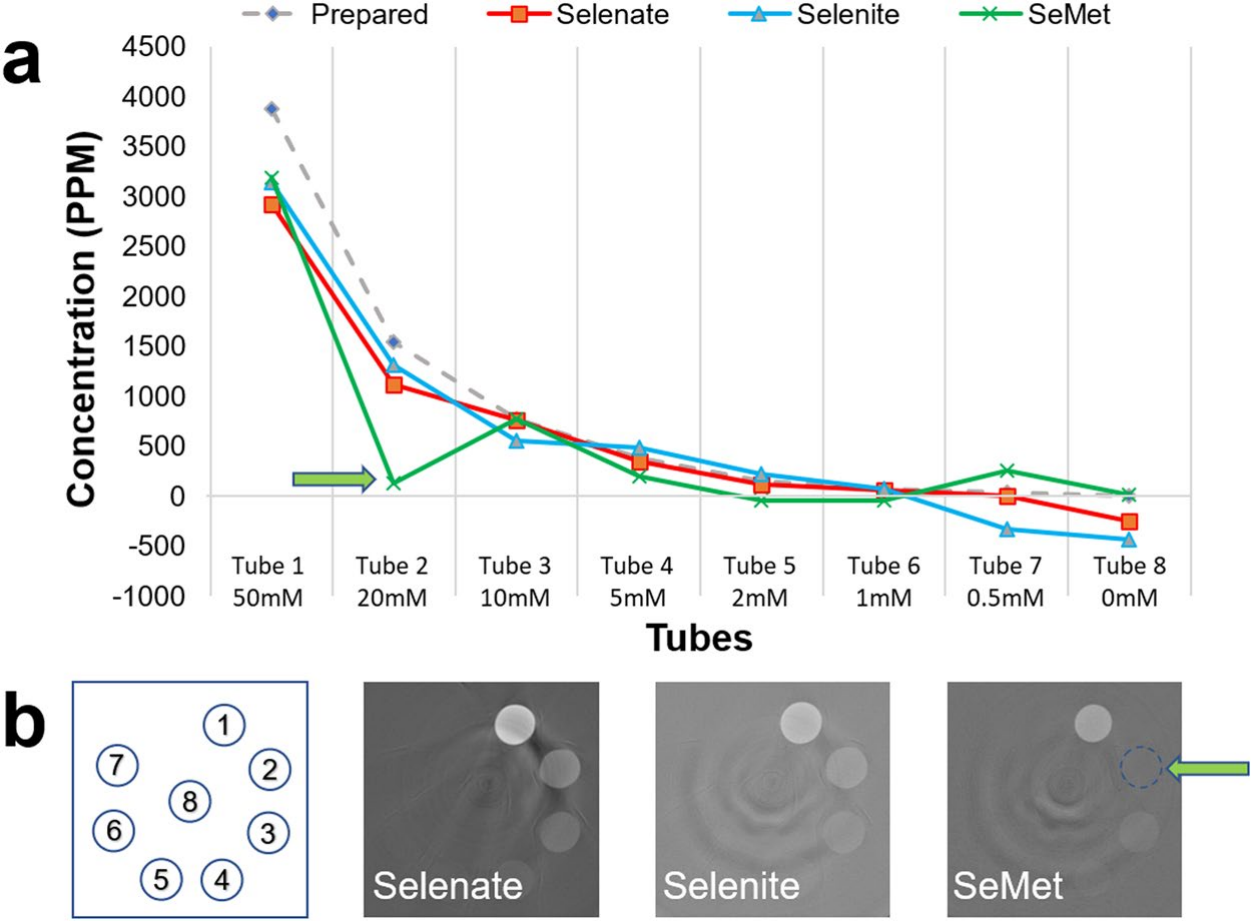
CT concentration sensitivity for Selenate, Selenite and SeMet. Figure a shows the graph of the measured vs. prepared solution concentration. The green arrow highlights an outlier value for SeMet where the solution leaked from the tube. The gray represents the prepared concentrations, (each solution sample was prepared with only one selenium species, and the gray bar only represents the species prepared in that sample), and colored bars represent measured concentrations.

To determine the concentration sensitivity of the system for Se speciation imaging, two sets of CT imaging experiments are reported. The first experiment is a detection limit test for the concentrations, the results of which are shown in Fig. 6. Overall, the detection limit of our speciation method is about 5 mM (400 ppm, 0.04% weight) in terms of the prepared concentration.

Other experiments were done to show that combinations of the three Se solutions could be distinguished but are not presented as they do not add significant additional information.

### Energy resolution

The energy resolution arises from (a) crystal lattice spacing compression/expansion, (b) source size effects, (c) the finite source to crystal distance, (d) intrinsic diffraction effect from the crystal, and (e) finite pixel size in the diffraction plane at the detector. Estimation of these contributions to the energy resolution are given in the Online Methods section. The total theoretical energy resolution is 2.52 eV. This resolution is dominated by the lattice plane expansion & compression (1.899 eV) and the intrinsic diffraction effect from the crystal (1.61 eV).

In order to independently determine the energy resolution of the monochromator, measurements and simulations were done. To assess the energy width associated with a single ray incident on the monochromator, a 10 µm diameter pinhole was placed in front of the crystal to prepare a small beam. The size of the diffracted beam at and near the Se K-edge was measured by an imaging detector (Hamamatsu AA60 + ORCA Flash 4.0) at a distance of 2.0 m from the monochromator. The contribution to the beam size at the detector is due to the Bragg plane rotation inside the wafer and, to a smaller extent, the size of beam through the pinhole (16 μm). The pinhole beam size is a combination, in quadrature, of the pinhole size (10 μm) and the bend magnet source size seen through the pinhole (12.5 μm) at the detector location.

**Figure 7:**
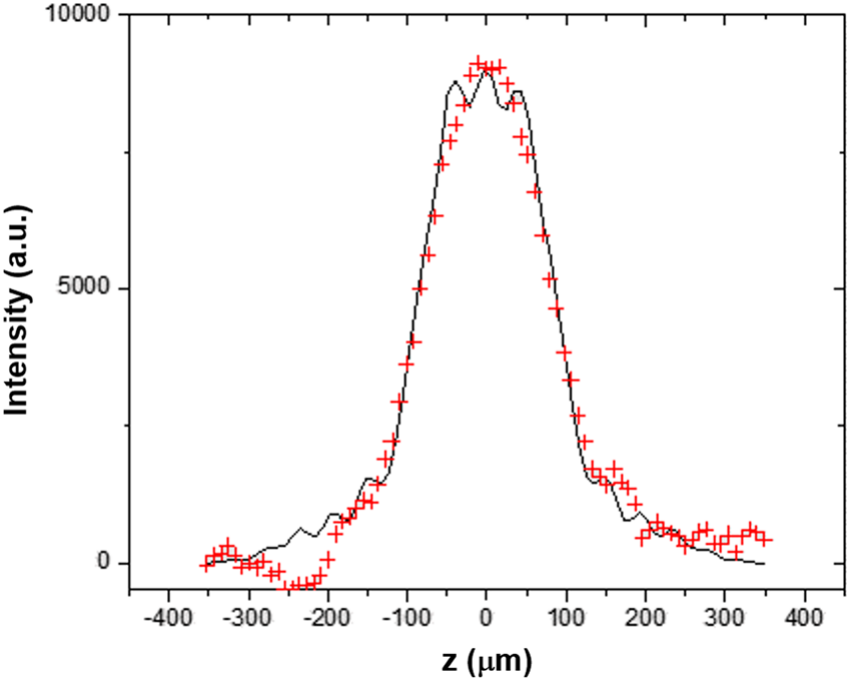
Measured (red crosses) and ray-tracing simulated peak (line) at the detector from a 10 µm diameter pinhole. Calculated peak width from sum of pinhole and Bragg planes rotation effect: 184 μm, ray-tracing result: 182 μm, measured: 181 μm.

Ray-tracing calculations were carried out with SHADOW^35^ to simulate the experimental set-up and the results accurately reproduced the beam size measurements (see Fig. 7), which demonstrate the validity of our model. The raytracing also shows that the peak at the detector has an energy width of 2.69 eV (More details are described in the Method section). This value matches the 2.52 eV width from the simple calculation as a sum of the various contributions.

## Discussion

In previous bent Laue EDXAS systems development, the coincidence of the geometric focus and the polychromatic focus^36^ has been considered to minimize the focal size^36–38^ which, in part, determines the spatial resolution of the imaging system. However, the method to optimize the energy dispersion and energy resolution has not been fully exploited. This paper describes an optimized bent Laue EDXAS system for speciation imaging of Se compounds. The optimization requires a specific lattice plane and asymmetric angle and crystal thickness, which is further described in the Online Method section. This results in the system being somewhat element specific.

Also, all functional EDXAS beamlines are adapted to use the horizontal dimension of the x-ray beam to provide wide energy range with horizontally diffracting bent crystal optics^10,11,39–42^. While this results in a small focused beam, it limits the ability to do full field imaging of larger specimens. The system presented here prepares a vertically dispersed beam with a line focus fully compatible with projection and computed tomography imaging. This allows for rapid screening of specimens.

For the system described here, the overall detection limit for Se compounds is about 5 mM (400 ppm, 0.04% weight) in terms of the prepared concentration (Fig. 6), which is better than typical low concentration limit (0.1% weight) expected for transmission XAS. The series of concentration measurements from CT showed overall agreement of 5% to the prepared values. The advantage in this case is demonstrated by the ability to do full field quantifiable 2D or 3D imaging in plant applications. As an example, some selenium accumulating species of *Astragalus* and *Stanleya* are able to contain selenium up to 1% dry weight^26,27,43^.

Referring to Fig. 5, there are two aspects that are of concern and require further attention. One, the systematic underestimation of the contrast values that were present in each of the tubes by approximately 20% at high concentration values (>7000 ppm). Also, there are some false positive and negative concentration values noted earlier. The reasons for these discrepancies are currently being investigated both in changes to our data analysis (non-negative least squares and iterative reconstruction methods) and to our monochromator (improved thermal cooling and better polishing to remove fine spatial structure in the imaging beam).

The energy resolution was modeled and measured to be about 2.69 eV, which is sufficient to visualize the near edge structure or speciation of the Se compounds. The system’s ability to do so for Se can be seen in Fig. 2a where the ‘white line’ for the three Se compounds can be clearly observed at different vertical (energy) locations. From the SHADOW simulation and pinhole test, we found 2.69 eV energy width. This is consistent with the 1.5 eV Gaussian blurring of measured data from the dedicated beamline, as well as with other works that model bent Laue EDXAS systems^11^.

Contributions to the energy resolution are dominated by the intrinsic diffraction effect from the crystal and the lattice plane expansion and compression, the latter being driven by the crystal thickness and the bending radius. The two contributions are quite close and indicate that the system is nearly optimal for the material/reflection chosen and the desired focal length of the EDXAS system. Other contributions to the energy resolution such as source size effect, beam divergence and the detector pixel size were found to be minimal.

For the examples shown, the effective pixel size was 13.6 μm parallel to the line focus and the estimated size at the focus was calculated to be 8 μm. The energy range covered could be as much as 500 eV, but in practice was limited to 100 ~ 200 eV to minimize dose. The delivered dose with the full 500 eV beam was measured at about 20.4 Gy/s with 250 mA ring current. A CT slice dataset with 900 angular steps covering 180 degrees took 12 minutes. The time limitation could be improved by reducing detector readout and motor motion time.

The data analysis method is based upon calculated or measured reference absorption coefficient values with a least square algorithm used to extract concentration or projected concentration values. The energy dependent absorption for all the compounds considered have differing near edge structure including the edge location. It is these differences that give the ability to independently determine from the imaging data, the amount of those materials in either projection or computed tomography. The measurements are interpreted against the reference data in a least square algorithm, to extract projected values for the three Se compounds and water from the data, such as shown in Fig. 3. This gives projected values for a single line where the focused beam intercepts the object. That line can form the basis of a set of 2D or projection images by scanning the object vertically through the beam, or a set of sinograms if the object is rotated. A CT example of a test object is shown in Fig. 4.

The ability to determine independent projected concentration (2D) or concentration (3D) values depends on having differences between the energy dependence of the absorption for each of the materials. In other words, over the energy range used by the system, the absorption coefficient for each of the materials must be linearly independent. For this reason, the method is not able to extract independent plastic or water values in the data presented. However, there is no limitation, other than computation, to the number of compounds that can be included in the analysis as long as they meet the linear independent condition. The same analysis method has been used to extract as many as six independent compounds^44^.

In summary, the reported imaging system (a) provides a wide horizontal imaging beam that is vertically energy dispersed allowing for wide field imaging applications, (b) uses an asymmetric bent Laue crystal monochromator which provides minimized focal size and optimized energy dispersion that is sufficient for Se speciation imaging, (c) has reasonable concentration sensitivity for rapid screening of specimens, and (d) provides quantifiable concentrations in 2D and 3D imaging.

## Methods

### Imaging system

As shown in the system layout (Fig. 1), the incident polychromatic beam from the synchrotron source strikes the bent Laue monochromator whose specific lattice planes diffract the imaging beam in the vertical plane. The lattice plane, asymmetry angle, and crystal thickness are chosen to match the imaging energy and desired angular energy dispersion properties. After the monochromator, the direct beam is stopped to prevent unnecessary scatter from reaching the object and detector. A lead shield is used at this location to allow the diffracted beam for this purpose. The bent crystal focuses the beam at the object location where translation and rotary stages position the object in the imaging beam and allows for linear scanning through the beam for projection imaging and rotation for computed tomography. The beam then diverges in the diffraction plane and after some drift distance, the beam is then intercepted and detected by an area or imaging detector.

The horizontal position (x) in the detector corresponds to the horizontal width of the beam and object, while the vertical position corresponds to the energy of the beam (E) defined by the monochromator and distance from the focus or the monochromator. The area detector thus measures the spectral transmission in an x-energy or x-E projection. Scanning the object vertically along y-axis (perpendicular to the central diffracted x-ray beam) gives an x-energy-y data set while rotating the object by an angle θ about the y-axis gives an x-energy-θ data set from which a slice can be reconstructed.

Using the Canadian Light Source (CLS) biomedical bend magnet beamline^45^, the photon rate per horizontal beam width and per milli-ampere storage ring current was measured at 1.07 × 10^9^ photons/(s • horizontal-mradian • mA) with an air-filled ionization chamber. As a comparison, a calculation of the vertically integrated photon rate for the same conditions using a double-crystal Si (1,1,1) Bragg monochromator was 1.18 × 10^9^ photons/(s • horizontal-mradian • mA), which is quite close to the measurement with our bent Laue monochromator. The dose rate was about 20.4 Gy/s with 250 mA ring current.

### Monochromator

For this work around the Se K-edge at 12.658 keV, 0.80 mm thick silicon wafers were purchased and were thinned to 0.30 mm for optimal Laue geometry diffraction performance (Addison Engineering, Inc). The wafers had a [2,2,4] surface normal and the (1,1,−1) reflection was used. The (1,1,−1) lattice planes were inclined 5 degrees from the surface normal in the diffraction plane which was chosen to provide nearly optimal energy dispersive properties^46^. The crystal wafer of the monochromator was bent to a 2.0 m radius which resulted in a 0.96 m focus downstream at the sample location.

**Figure 8:**
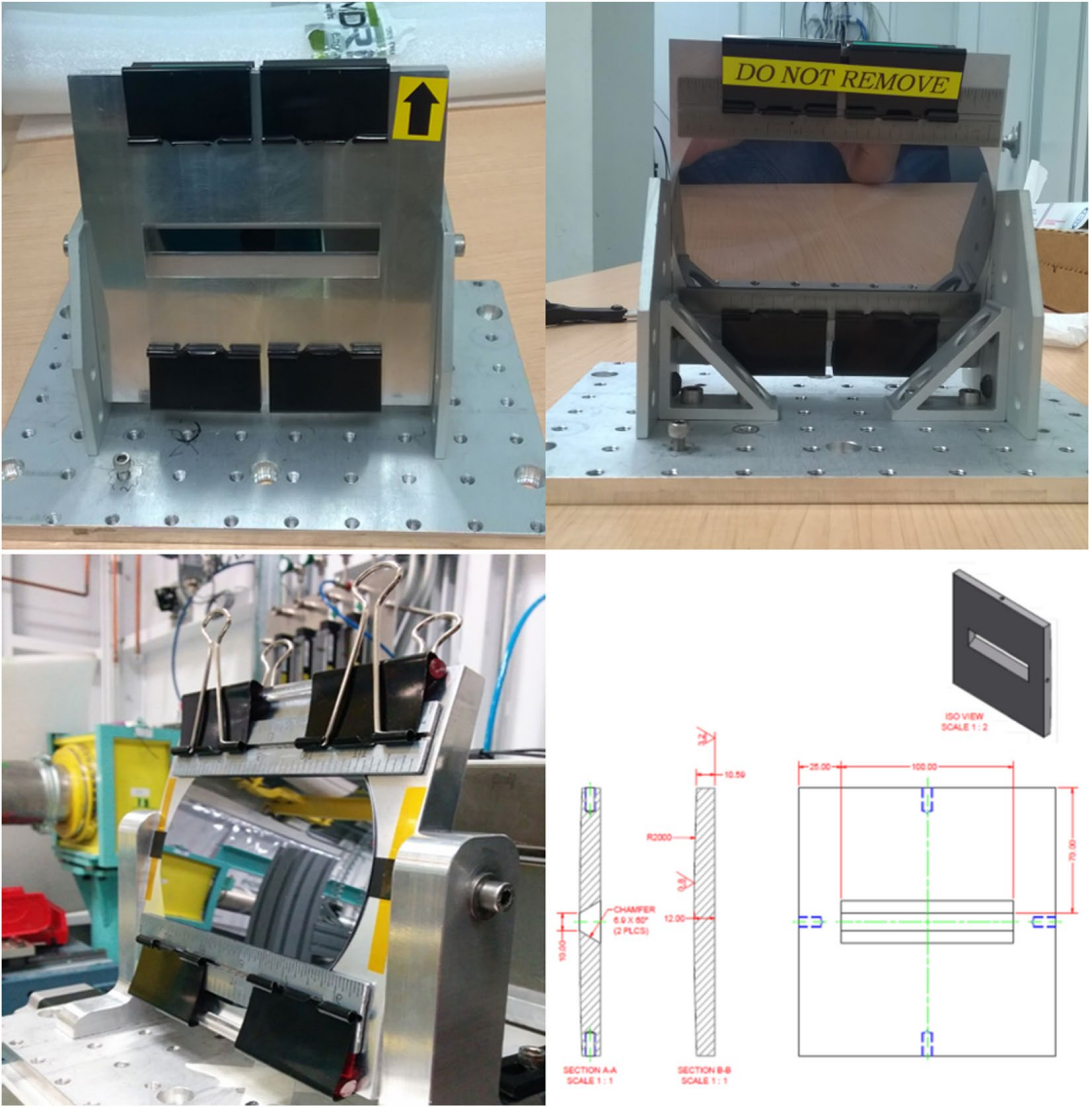
Bent Laue monochromator. The designed radius of the cylindrical side of the frame bender is 2 m.

The frame bender was designed to be cylindrical on one side with a radius of 2.0 m, and flat on the other side. The window through the frame bender is narrower on the cylindrical side and wider on the other side, allowing the emergent beam to go through the bender after diffraction. The crystal was fixed on to the cylindrical side of the frame bender with stainless steel ruler and binder clips. (Fig. 8)

The vertical size of the incident beam on the crystal give an energy range of about 500 eV. However, given the vertical distribution of the intensity, the energy range was typically limited to about half that value. The detector was approximately 2.4 m downstream from the monochromator (Fig. 1).

### Bent Laue energy dispersion geometry (magic condition)

**Figure 9:**
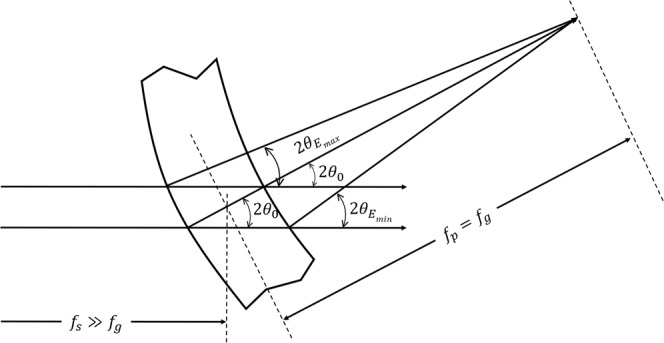
Bent Laue geometry crystal that meets the ‘magic condition’ has overlapped geometrical focus (${f}_{g}$) and polychromatic focus (${f}_{p}$).

In the Laue or transmission geometry with a bent crystal plate, each incident x-ray beam that strikes the crystal undergoes two overall effects that influence the focal and energy dispersive properties. These two effects are shown in Fig. 9. Each incident polychromatic x-ray that traverses the crystal in a focusing geometry will diffract in depth through the crystal. Typically, these will result in a bundle of diffracted x-rays that exit the crystal plate on a converging trajectory that will focus at the so-called “polychromatic” or “single-ray” focus designated as ${f}_{p}$ in Fig. 9. Other incident rays that are parallel but displaced in the diffraction plane will form a focus due to the overall bending of the crystal (${f}_{g}$ in Fig. 9). This is analogous to the focusing of a lens and the formation of a geometric focus. Typically, these two foci will occur at different distances downstream of the crystal. The energy of each diffracted beam is mostly defined (aside from crystal expansion and compression due to bending) by the scattering angle of the beams or the 2*θ* angle (2*θ* in Fig. 9). Using Bragg’s law, these scattering angles are directly related to the diffracted beam’s wavelength or energy using ${\rm{\lambda }}=2d\,\sin \,{\rm{\theta }}$. By inspection of the figure, if each ray that exits the crystal arrives at a single focus (${f}_{p}={f}_{g}$), then it will have a unique wavelength or energy irrespective of the origin of the ray (either from the geometric effects of the lattice curvature or diffraction in depth through the crystal or polychromatic focal effects). Again, this is true only if the lattice is not compressed or expanded on bending. Thus, if the two focal lengths are matched, then it should be possible to (1) have a very small focal spot and (2) very good energy dispersive properties. This is the geometry used for the bent Laue energy dispersive monochromator and is what we call the ‘magic condition’^15,47^.

### The ‘magic condition’

As noted for minimal focus and optimal energy dispersive properties, both the geometric and polychromatic foci should be co-located. The geometric focus is defined by^36^:

1 $$\frac{\cos (\chi \mp {\theta }_{B})}{{f}_{g}}-\frac{\cos (\chi \pm {\theta }_{B})}{{f}_{s}}=\frac{2}{R}$$

where $\chi$ is the asymmetry angle of the lattice planes, ${\theta }_{B}$ is the Bragg angle, *f*_g_ is the geometric focus distance, *f*_s_ is the source to the crystal distance and *R* is the bend radius of the crystal. The signs identify the relationship between the entrance and exit beams, the lattice planes and the bend radius.

The polychromatic focus is defined by^15^:

2 $${f}_{p}=\pm \frac{R\,\sin \,2{{\rm{\theta }}}_{B}}{2\,\sin ({\rm{\chi }}\pm {{\rm{\theta }}}_{B})+(1+{\rm{\nu }})\sin \,2{\rm{\chi }}\,\cos ({\rm{\chi }}\pm {{\rm{\theta }}}_{B})}$$

where ${\rm{\nu }}$ is the Poisson ratio, and the sign convention is the same as in Eq. (1).

The condition for achieving a small focus is achieved when (1) the upper sign is used and (2) the geometric focus, ${f}_{g}$, and polychromatic focus, ${f}_{p}$, match. The solution for the focal match is best done numerically. The solution will be independent of the bend radius and will relate the Bragg angle to the asymmetry angle. A simple solution can be found if one assumes that the source to crystal distance is large (${f}_{s}\gg {f}_{g}$) and the photon energy is high (${\theta }_{B}$ ≪ 1) which leads to:

3 $${\rm{\chi }}\approx \frac{{{\rm{\theta }}}_{B}}{2+{\rm{\nu }}}$$

which indicates that the asymmetry angle choice needs to be small for the magic condition to be met.

A [2,2,4] type wafer was used as the monochromator crystal. The (1,1,−1) lattice planes were used for the diffraction planes for the monochromator. With no asymmetry angle the (1,1,−1) lattice planes will be perpendicular to the wafer. However, the wafer was cut with a 5 degree offset towards the [2,−2,0] direction, which results in a 5 degree asymmetry angle of the (1,1,−1) plane. Based on the discussion above, the focal matching condition of the asymmetric angle would be 4.47 degrees. This mismatch of the asymmetry angle from the ideal condition will result in focal blurring and minimal effect on the energy dispersive properties, which will now be discussed.

#### Beam size at focus

For the conditions given, with an asymmetry angle of 5 degree for the (1,1,−1) lattice planes, there is a mismatch from the calculated 4.47 degrees value needed to match the two foci. This mismatch of 0.53 degrees will result in an estimated beam size at the geometric focus of 5.87 μm. The beam size at the focus will also be affected by the source size which for the BMIT bend magnet is 118 μm FWHM. The de-magnified source size will then be 5.12 μm (source to crystal distance of 22 m and crystal to geometric focus 0.96 m). The combination of the two contributions results in a focus of 7.79 μm when added in quadrature.

#### Estimated depth of field

The detector used for imaging had a pixel size of 13.6 μm. A conservative estimate of the spatial resolution would be no less than about 30 μm. The energy range used in the data analysis corresponds to a vertical beam size of about 0.5 mm which focuses about 1 m from the crystal. Thus, the distance from the approximate 8 μm focus where the beam size is equal to the spatial resolution is about 4.4 mm. Therefore, the estimated depth of field is about 8.8 mm.

#### Energy resolution with the magic condition

The magic condition results in every ray diffracted within the crystal arriving at the same focus. The energy spread of each ray going towards that focus (focal ray) will be set by the range of energies that occur from where the ray originates at the entrance side of the crystal to where it exits on the other side. Since every incident beam ray that intersects a focal ray will have the scattering angle, $2\theta$, then it also has the same Bragg angle, $\theta$. Under this condition, the spread of wavelengths in the focal ray will be set only by the spread of lattice plane spacings along the focal ray. Specifically, in terms of energy wavelength or bandwidth

$$\frac{{\rm{\delta }}{\rm{\lambda }}}{{\rm{\lambda }}}=\frac{{\rm{\delta }}d}{d}=\frac{{\rm{\delta }}E}{E}$$

where ${\rm{\lambda }}$ is the x-ray wavelength, $d$ is the lattice plane spacing, and $E$ is the x-ray energy. Also, the equation holds for the magnitude of the values. For a symmetric Laue type crystal bent to a cylinder, the lattice plane spacing on the entrance and exit surfaces can be easily calculated with

$$\frac{{\rm{\delta }}d}{d}=\frac{T}{R}$$

where $R$ is the bending radius of the crystal, and $T$ is the thickness of the bent crystal, which can be calculated with the nominal thickness ${T}_{0}$ in absence of bend, and the bending radius $R$^47^

$$T=\frac{R}{{\rm{\nu }}}\cdot ({e}^{\frac{{\rm{\nu }}{T}_{0}}{R}}-1)$$

Therefore, the energy spread along the focal ray is

$$\frac{{\rm{\delta }}E}{E}=({e}^{\frac{{\rm{\nu }}{T}_{0}}{R}}-1)/{\rm{\nu }}$$

which is determined by the thickness of the crystal ${T}_{0}$ and the bending radius $R$. For an asymmetric bent Laue crystal, the lattice spacing is affected by the asymmetry angle ${\rm{\chi }}$, but the effect is small when ${\rm{\chi }}$ is small. Given the parameters for our crystal, ${\rm{\delta }}E/E=1.50\times {10}^{-4}$. At the Se K-edge energy 12.658 keV, the energy resolution, $\Delta {E}_{1}$, for the single ray energy spread is 1.899 eV. $\Delta {E}_{1}$ can be improved through two approaches: decreasing the crystal thickness reduces the reflectivity of the bent crystal while allowing more transmission; in addition, increasing the bending radius reduces the energy bandwidth of the bent crystal.

There are four additional contributions to the total energy resolution of the monochromator. (i) The source size angular spread at the crystal. At 22 m distance from the source, the angular spread of the source, $\Delta {\theta }_{s}$, is 5.364 ${\rm{\mu }}rad$, which results in $\Delta {E}_{2}\,=\,0.429\,\,$eV at the Se K edge energy 12.658 keV. (ii) The intrinsic Darwin width of the curved Laue crystal, which results in Δ*E*_3_ = 1.609 eV. (iii) The vertical divergence from the finite source distance, which results in $\Delta {E}_{4}=\,0.338$ eV. (iv) the pixel size of the detector. With the 13.6 $\mu m$ pixel size and 1460 mm focus-to-detector distance, the contribution $\Delta {E}_{5}=0.376\,{\rm{eV}}$. The total energy resolution, $\Delta E$, can be calculated with

$$\Delta E=\sqrt{\Delta {E}_{1}^{2}+\Delta {E}_{2}^{2}+\Delta {E}_{3}^{2}+\Delta {E}_{4}^{2}+\Delta {E}_{5}^{2}}=2.576\,eV$$

### Energy resolution measurements

Measurements were made in order to independently determine the energy resolution of the monochromator.

A 10 μm diameter pinhole in front of the crystal was used to prepare a small beam onto the monochromator, and the size of the beam was measured at the detector. The contribution to the beam size at the detector is due to the Bragg plane rotation inside the wafer

$$2\cdot {{\rm{\theta }}}_{{\rm{rotation}}}\cdot 2.41{\rm{m}}-\frac{{\rm{T}}\cdot \,\sin \,2{\rm{\theta }}}{\cos ({\rm{\chi }}+{\rm{\theta }})}=168\,{\rm{\mu }}m$$

and, to a smaller extent, the size of the pinhole (16 mm). Ray-tracing calculations (SHADOW^35^) were conducted to simulate the experimental set-up and the results accurately reproduced the measurements (see Fig. 7), which demonstrate the validity of our model.

**Figure 10:**
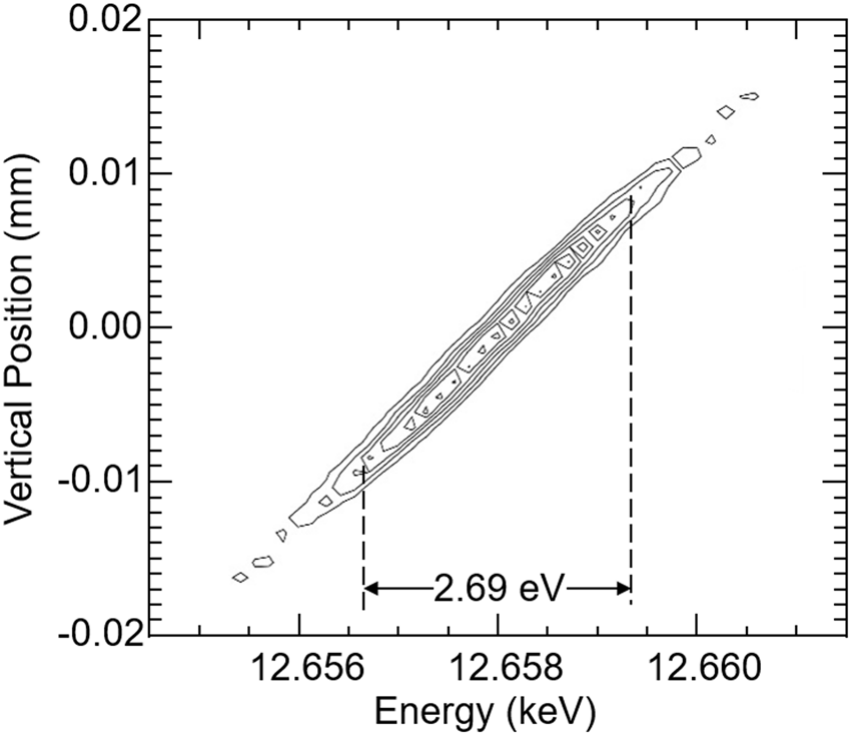
Ray tracing results for a 10 μm pinhole in front of the crystal showing the energy resolution and the vertical size (sizes are shown in cm).

**Figure 11:**
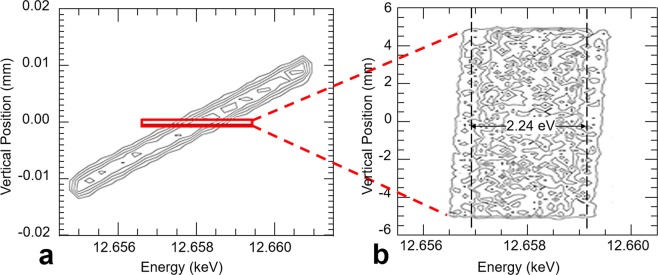
If we simulate a larger beam compared with Fig. 10, we obtain the energy vs vertical beam location dependence as shown here (left). We then choose the rays within a 10 μm vertical window to show the energy resolution at each detector pixel.

The raytracing also shows that the peak at the detector has an energy width of 2.69 eV as shown in Fig. 10. This value matches a simple calculation for the peak energy as a sum of the ΔE due to the Bragg planes rotation and lattice d-space compression (2.41 eV) and the energy contribution from the pinhole size (0.43 eV), for a total of 2.84 eV. The energy vs pinhole vertical sizes are shown in Fig. 11. In Shadow’s coordinate system the vertical beam position is inverted.

The comparison in Fig. 7 demonstrates that our ray-tracing simulation is correct and hence the simulation for the energy resolution using our model should also be correct. We then simulated a larger beam (2 mm in the vertical direction) and from it we chose the beam in a 10 ${\rm{\mu }}m$ window, simulating the detector pixel size. The result is 2.24 eV as shown Fig. 11. (Note: Darwin Widths of (1,1,1) and (2,2,0) at 12.67 keV are 20.4 and 15.1 $\,{\rm{\mu }}m$, compared to the relative peak width for our bent (1,1,1) of 30.1 $\,{\rm{\mu }}m$.)

### Analysis method

At a specific $x$ location, assume that each vertical line in the detector is a measurement of the number of photons transmitted through the object as a function of energy, $N({E}_{i})$, with an incident photon count to the object of, ${N}_{0}({E}_{i})$. The detector pixels along each vertical line will divide the energy range into *n* different energies values (1 ≤*i* ≤ *n*). Also, assume that the object at that x location is composed of *m* different materials indexed by *j*, whose mass attenuation coefficients are $({(\frac{{\rm{\mu }}}{{\rm{\rho }}})}_{ji}{E}_{i})$, densities of, $\,{\rho }_{j}$, and thicknesses of, $\,{t}_{j}$, for the selenium and matrix materials.

4 $$N({E}_{i})={N}_{0}({E}_{i}){e}^{-\mathop{\sum }\limits_{j=1}^{m}{(\frac{{\rm{\mu }}}{{\rm{\rho }}})}_{j}({E}_{i}){{\rm{\rho }}}_{j}{t}_{j}}\,or\,{N}_{i}={N}_{{0}_{i}}{e}^{-\mathop{\sum }\limits_{j=1}^{m}{(\frac{{\rm{\mu }}}{{\rm{\rho }}})}_{ji}{{\rm{\rho }}}_{j}{t}_{j}}$$

The *i* index corresponds to the energy of photons detected across the vertical extent of the beam on the detector (*i* = 1 to *n* energies) and the *j* index in the exponent corresponds to the various materials responsible for attenuating the transmitted beam through the object. The right side of Eq. (1) is a short-hand version to simplify subsequent equations. The attenuation characteristics of the contrast and matrix materials $({(\frac{{\rm{\mu }}}{{\rm{\rho }}})}_{ji})$ can be measured, tabulated or modeled.

Equation (4) can be recast by taking a negative logarithm to give a set of *n* linear equations with *m* unknowns,

5 $${r}_{i}=-\,\mathrm{ln}(\frac{{N}_{i}}{{N}_{{0}_{i}}})=\mathop{\sum }\limits_{j=1}^{m}{(\frac{{\rm{\mu }}}{{\rm{\rho }}})}_{ji}{{\rm{\rho }}}_{j}{t}_{j},1\le i\le n$$

Now we need to find the projected values (*ρ*_*j*_*t*_*j*_) that lead to the best fit to the measured values (*r*_i_). A least-squared approach is used^14,48^ and results in

6 $$[\begin{array}{c}{{\rm{\rho }}}_{1}{t}_{1}\\ \vdots \\ {{\rm{\rho }}}_{j}{t}_{j}\\ \vdots \\ {{\rm{\rho }}}_{m}{t}_{m}\end{array}]={[\begin{array}{ccccc}n{(\frac{{\rm{\mu }}}{{\rm{\rho }}})}_{\langle 11\rangle } & \ldots  & n{(\frac{{\rm{\mu }}}{{\rm{\rho }}})}_{\langle 1j\rangle } & \cdots  & n{(\frac{{\rm{\mu }}}{{\rm{\rho }}})}_{\langle 1m\rangle }\\ \vdots  &  & \vdots  &  & \vdots \\ n{(\frac{{\rm{\mu }}}{{\rm{\rho }}})}_{\langle j1\rangle } & \ldots  & n{(\frac{{\rm{\mu }}}{{\rm{\rho }}})}_{\langle jj\rangle } & \ldots  & n{(\frac{{\rm{\mu }}}{{\rm{\rho }}})}_{\langle jm\rangle }\\ \vdots  &  & \vdots  &  & \vdots \\ n{(\frac{{\rm{\mu }}}{{\rm{\rho }}})}_{\langle m1\rangle } & \ldots  & n{(\frac{{\rm{\mu }}}{{\rm{\rho }}})}_{\langle mj\rangle } & \ldots  & n{(\frac{{\rm{\mu }}}{{\rm{\rho }}})}_{\langle mm\rangle }\end{array}]}^{-1}[\begin{array}{c}\sum _{i}({(\frac{{\rm{\mu }}}{{\rm{\rho }}})}_{1i}{r}_{i})\\ \vdots \\ \sum _{i}({(\frac{{\rm{\mu }}}{{\rm{\rho }}})}_{ji}{r}_{i})\\ \vdots \\ \sum _{i}({(\frac{{\rm{\mu }}}{{\rm{\rho }}})}_{mi}{r}_{i})\end{array}]$$

where

7 $${\frac{{\rm{\mu }}}{{\rm{\rho }}}}_{\langle kl\rangle }\equiv \frac{1}{n}\mathop{\sum }\limits_{i=1}^{n}[{(\frac{{\rm{\mu }}}{{\rm{\rho }}})}_{ki}{(\frac{{\rm{\mu }}}{{\rm{\rho }}})}_{li}]={\frac{{\rm{\mu }}}{{\rm{\rho }}}}_{\langle kl\rangle }$$

Thus, the task of determining the projected material values is reduced to determining the mass attenuation weighted values using the measured transmission values from Eq. (5) and the inverse of the mass attenuation matrix in Eq. (6). The components in that matrix are “squares” of the mass attenuation coefficients averaged over the energy range of the measurements as defined in Eq. (7). For simplicity, the inverse of the matrix will not be shown here but is easily evaluated numerically by computer.

### Sample preparation

Sample holders for proof-of-principle experiments comprised 4 nylon tubes in circle, with a seedpod of *Astragalus bisulcatus* in the center. The bundle was then fixed in a disposable plastic liquid dropper to prevent unexpected sample position movement while we adjusted the stage. Every nylon tube was sealed on both ends to contain certain solution samples as described in the Result section.

**Figure 12:**
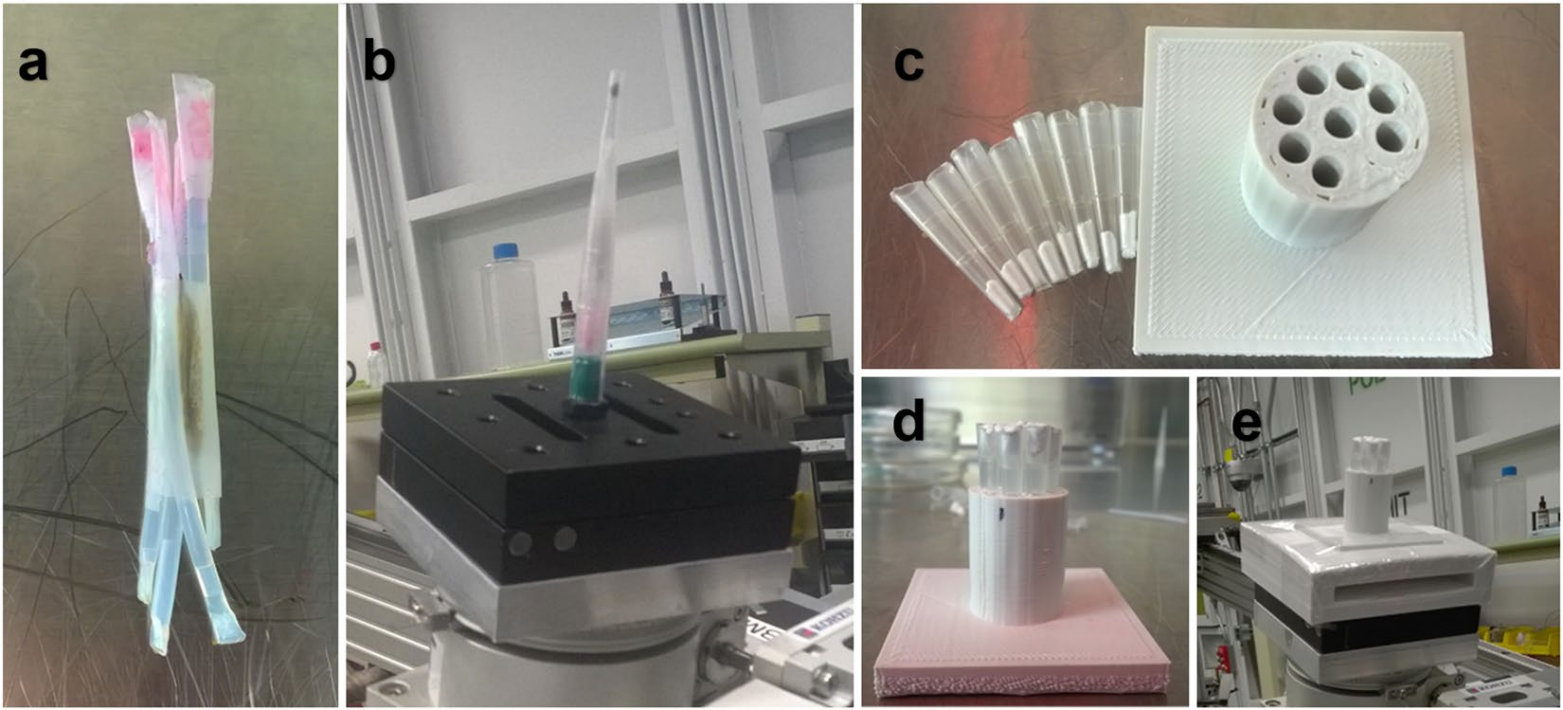
Samples and sample holders. (**a**) Solution samples in nylon tubes surrounding an *Astragalus bisulcatus* seedpod. (**b**) Nylon tubes and the seedpod fixed in a plastic liquid dropper on the imaging stage. (**c**) Top view of sample holder base and solution containers for concentration sensitivity tests. (**d**) Side view of sample holder for concentration sensitivity tests. (**e**) Sample holder for concentration sensitivity tests on the imaging stage.

The sample holder for concentration sensitivity tests is a 3D printed cylinder with 8 hollows, designed with SketchUp. Pipette tips sealed on both ends are used as containers for solution samples and were placed in the hollows of the holder during experiment. Sample holders and containers are shown in Fig. 12.

### Data collection

All experiments were performed at the BMIT BM beamline at the Canadian Light Source (CLS) at the University of Saskatchewan. The imaging sample was placed at the focus and the prepared beam was then transmitted through the sample into a high-resolution x-ray detector (Hamamatsu AA-60 with ORCA Flash 4.0, which has an effective pixel size of 13.6 µm), forming a 2-dimensional image with horizontal position in the sample along the x-axis and a range of energy along the y-axis. The distances are 22 m from synchrotron source to the bent crystal, 0.96 m from the bent crystal to the imaging sample, and 1.34 m from sample to the detector. Due to the high heat load on the crystal and the resulting distortion of its shape, the beam was allowed to stabilize for about 40 minutes for each sample before imaging. A water cooler to stabilize the temperature of the monochromator in shorter time length was designed and used for subsequent experiments. As a result, stabilization time was shortened to 10 ~ 15 minutes.

The image produced in one projection represents the absorption spectrum of one horizontal position in the imaged sample. To produce 2-dimensional projection images, the samples were vertically scanned by moving the sample stage to capture a series of energy-spatial images, each representing a single vertical position in the sample. To produce CT slices, the samples were rotated through 180° with 900 energy-spatial images captured at regular intervals; the CT scan for one slice generally takes 12 to 14 minutes.

## References

[1] Lobinski R, Moulin C, Ortega R (2006). Imaging and speciation of trace elements in biological environment. Biochimie.

[2] Wu B, Becker JS (2012). Imaging techniques for elements and element species in plant science. Metallomics.

[3] Buschert R, Giardina MD, Merlini A, Balerna A, Mobilio S (1988). Laboratory EXAFS in a dispersive mode. J. Appl. Crystallogr..

[4] Newton Mark A., Dent Andrew J. (2013). Energy-Dispersive EXAFS: Principles and Application in Heterogeneous Catalysis. In-situ Characterization of Heterogeneous Catalysts.

[5] Phizackerley RP (1983). An energy-dispersive spectrometer for the rapid measurement of X-ray absorption spectra using synchrotron radiation. J. Appl. Crystallogr..

[6] Dartyge E, Fontaine A, Tourillon G, Cortes R, Jucha A (1986). X-ray absorption spectroscopy in dispersive mode and by total reflection. Phys. Lett. A.

[7] Mathon Olivier, Kantor Innokenty, Pascarelli Sakura (2016). Time-Resolved XAS Using an Energy Dispersive Spectrometer: Techniques and Applications. X-Ray Absorption and X-Ray Emission Spectroscopy.

[8] Pascarelli Sakura, Mathon Olivier (2016). Energy Dispersive XAS. XAFS Techniques for Catalysts, Nanomaterials, and Surfaces.

[9] Hagelstein M (1995). XAFS with an energy-dispersive Laue monochromator. Phys. B Condens. Matter.

[10] Ouyang ZW (2009). Insulator–metal phase transition of Pr _0.6_ Ca _0.4_ MnO _3_ studied by x-ray absorption spectroscopy in pulsed magnetic fields. J. Phys. Condens. Matter.

[11] Pascarelli S (2016). The Time-resolved and Extreme-conditions XAS (TEXAS) facility at the European Synchrotron Radiation Facility: the energy-dispersive X-ray absorption spectroscopy beamline ID24. J. Synchrotron Radiat..

[12] Guigay JP, Ferrero C (2016). Dynamical focusing by bent, asymmetrically cut perfect crystals in Laue geometry. Acta Crystallogr. Sect. A Found. Adv..

[13] Borrmann G. (1959). Röntgenwellenfelder. Beiträge zur Physik und Chemie des 20. Jahrhunderts.

[14] Zhu Y (2014). Spectral K-edge subtraction imaging. Phys. Med. Biol..

[15] Martinson M, Samadi N, Bassey B, Gomez A, Chapman D (2015). Phase-preserving beam expander for biomedical X-ray imaging. J. Synchrotron Radiat..

[16] Samadi, N. *et al*. An energy dispersive bent Laue monochromator for K-edge subtraction imaging. in *Proceedings of the 12th international conference on synchrotron radiation instrumentation (SRI2015)* (ed. Shen, Q & Nelson, C.) **1741**, 040004 (Amer Inst Physics, 2016).

[17] Montes-Bayón M (2002). Initial Studies of Selenium Speciation in Brassica juncea by LC with ICPMS and ES-MS Detection:  an Approach for Phytoremediation Studies. Anal. Chem..

[18] Bañuelos GS (2011). Selenium Accumulation, Distribution, and Speciation in Spineless Prickly Pear Cactus: A Drought- and Salt-Tolerant, Selenium-Enriched Nutraceutical Fruit Crop for Biofortified Foods. Plant Physiol..

[19] Pickering IJ, Prince RC, Salt DE, George GN (2000). Quantitative, chemically specific imaging of selenium transformation in plants. Proc. Natl. Acad. Sci..

[20] Combs GF (2001). Selenium in global food systems. Br. J. Nutr..

[21] Clark LC (1996). Effects of selenium supplementation for cancer prevention in patients with carcinoma of the skin. A randomized controlled trial. Nutritional Prevention of Cancer Study Group. JAMA.

[22] Ellis DR, Salt DE (2003). Plants, selenium and human health. Curr. Opin. Plant Biol..

[23] Whanger PD (2004). Selenium and its relationship to cancer: an update. Br. J. Nutr..

[24] Rayman MP, Infante HG, Sargent M (2008). Food-chain selenium and human health: spotlight on speciation. Br. J. Nutr..

[25] Tsubura A, Lai Y-C, Kuwata M, Uehara N, Yoshizawa K (2011). Anticancer Effects of Garlic and Garlic-derived Compounds for Breast Cancer Control. Anticancer. Agents Med. Chem..

[26] Davis A. M. (1972). Selenium Accumulation in a Collection of Atriplex Species. Agronomy Journal.

[27] Davis A. M. (1986). Selenium Uptake in Astragalus and Lupinus Species1. Agronomy Journal.

[28] Freeman JL (2006). Spatial imaging, speciation, and quantification of selenium in the hyperaccumulator plants Astragalus bisulcatus and Stanleya pinnata. Plant Physiol..

[29] Tse JJ, Gallego-Gallegos M, Franz ED, Liber K, Pickering IJ (2012). Selenium speciation and localization in chironomids from lakes receiving treated metal mine effluent. Chemosphere.

[30] Carey A-M (2012). A review of recent developments in the speciation and location of arsenic and selenium in rice grain. Anal. Bioanal. Chem..

[31] Valdez Barillas JR (2012). Selenium Distribution and Speciation in the Hyperaccumulator Astragalus bisulcatus and Associated Ecological Partners. Plant Physiol..

[32] Weekley CM (2014). XAS and XFM studies of selenium and copper speciation and distribution in the kidneys of selenite-supplemented rats. Metallomics.

[33] Pickering IJ, George GN, Van Fleet-Slalder V, Chasteen TG, Prince RC (1999). X-ray absorption spectroscopy of selenium-containing amino acids. J. Biol. Inorg. Chem..

[34] Etschmann BE (2014). Speciation mapping of environmental samples using XANES imaging. Environ. Chem..

[35] Sanchez del Rio M, Canestrari N, Jiang F, Cerrina F (2011). SHADOW3: a new version of the synchrotron X-ray optics modelling package. J. Synchrotron Radiat..

[36] Schulze C (1998). Microfocusing of Hard X-rays with Cylindrically Bent Crystal Monochromators. J. Synchrotron Radiat..

[37] Suortti, P., Lienert, U. & Schulze, C. Bent crystal optics for high energy synchrotron radiation. In *The 17th international conference on x-ray and inner-shell processes* **389**, 175–192 (ASCE, 1997).

[38] Pascarelli S, Mathon O, Aquilanti G (2004). New opportunities for high pressure X-ray absorption spectroscopy using dispersive optics. J. Alloys Compd..

[39] Cezar JC (2010). Energy-dispersive X-ray absorption spectroscopy at LNLS: investigation on strongly correlated metal oxides. J. Synchrotron Radiat..

[40] Bhattacharyya D, Poswal AK, Jha SN, Sangeeta, Sabharwal SC (2009). X-ray absorption spectroscopy of PbMoO4 single crystals. Bull. Mater. Sci..

[41] Baudelet F (2011). ODE: a new beam line for high-pressure XAS and XMCD studies at SOLEIL. High Press. Res..

[42] Poo-arporn Y (2012). Time-resolved XAS (Bonn-SUT-SLRI) beamline at SLRI. J. Synchrotron Radiat..

[43] Shrift A, Virupaksha TK (1965). Seleno-amino acids in selenium-accumulating plants. Biochim. Biophys. Acta - Gen. Subj..

[44] Bassey B (2016). Multiple energy synchrotron biomedical imaging system. Phys. Med. Biol..

[45] Wysokinski TW (2007). Beamlines of the biomedical imaging and therapy facility at the Canadian light source—Part 1. Nucl. Instruments Methods Phys. Res. Sect. A Accel. Spectrometers, Detect. Assoc. Equip..

[46] Zhang, H. *Imaging Dilute Contrast Materials in Small Animals Using Synchrotron Light* (2009).

[47] Qi, P., Shi, X., Samadi, N. & Chapman, D. Focusing and energy dispersion properties of a cylindrically bent asymmetric Laue crystal. In *Advances in X-Ray/EUV Optics and Components XIV* (eds. Morawe, C., Khounsary, A. M. & Goto, S.) **11108E**, 12 (SPIE, 2019).

[48] Kozul, N., Davis, G. R. & P Anderson, J. C. E. Elemental quantification using multiple-energy x- ray absorptiometry. *Meas. Sci. Technol* **10** (1999).

